# Perovskite Photodetectors Based on p-i-n Junction With Epitaxial Electron-Blocking Layers

**DOI:** 10.3389/fchem.2020.00811

**Published:** 2020-09-15

**Authors:** Yubing Xu, Xin Wang, Yuzhu Pan, Yuwei Li, Elias Emeka Elemike, Qing Li, Xiaobing Zhang, Jing Chen, Zhiwei Zhao, Wei Lei

**Affiliations:** ^1^School of Electronic Science and Engineering, Joint International Research Laboratory of Information Display and Visualization, Southeast University, Nanjing, China; ^2^Chemistry Department of North West University, Potchefstroom, South Africa

**Keywords:** photodetectors, self-powered, perovskite single crystals, epitaxial growth, X-ray detection

## Abstract

Organic-inorganic hybrid perovskite single crystals (PSCs) have been emerged as remarkable materials for some optoelectronic applications such as solid-state photodetectors, solar cells and light emitting diodes due to their excellent optoelectronic properties. To decrease the dark current, function layers based on spin-coating method are frequently requested for intrinsic PSCs to block the injected current by forming energy barrier. However, the amorphous function layers suffer from small carrier mobility and high traps density, which limit the speed of the photoelectric response of perovskite devices. This work supposes to grow thick MAPbBr_3_ and MAPbI_3_ mono-crystalline thin films on the surface of intrinsic MAPbBr_2.5_Cl_0.5_ PSCs substrate by a heteroepitaxial growth technique to act as electron-blocking layers. Meanwhile, C60 and [6,6]-phenyl-C61-butyric acid methyl ester (PCBM) layers are deposited on the opposite surface of substrate PSCs by spin-coating method to block injected holes. This Au-MAPbI_3_-MAPbBr_3_-MAPbBr_2.5_Cl_0.5_PSCs-C60-PCBM-Ag heterostructure can be used as excellent X-ray photodetector (XPD) due to its low dark current density of 6.97 × 10^−11^ A cm^−2^ at −0.5 V bias, high responsivity of 870 mA/W at −100 V bias and X-ray sensitivity as high as 59.7 μC mGy^−1^ cm^−2^ at −50 V bias.

**Graphical Abstract:**
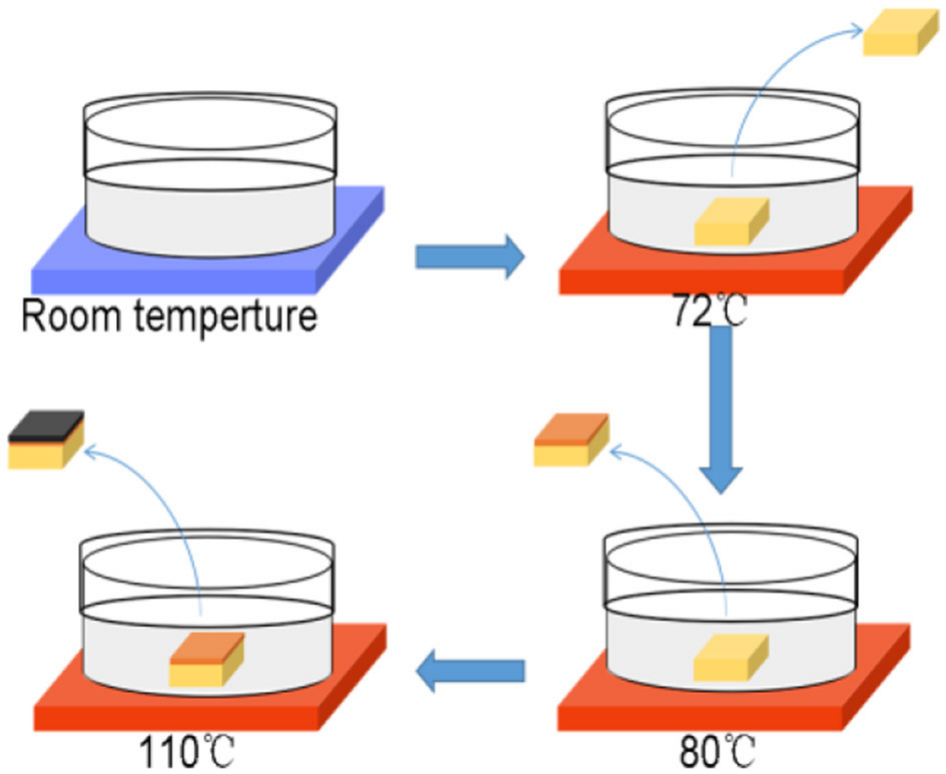
Solution-processed epitaxial growth of electron-blocking layers.

## Introduction

The last few years have witnessed the intensive investigation on Organic-inorganic hybrid perovskite single crystals (OIHPSCs) by many researchers due to their advantages of high photons absorption coefficients, high charges mobility and adjustable band gaps (Dou et al., 2014). Besides, self-powered photodetectors based on p-n junction and heterostructure can work stably without the applied voltage, which results in their improvement. Moreover, solution-processed optoelectronic devices have shown lower cost compared to those fabricated by vacuum deposition (Dong et al., 2015a; Miao and Zhang, 2019). Especially, OIHPSCs could be grown from solution with bulk size up to centimeter-scale and have shown attractive potential in X-ray detection and gamma ray detection (Wei et al., 2016). In that case, self-powered photodetectors based on solution-processed OIHPSCs tend to have better performance.

The common chemical formulas of OIHP are MAPbX_3_ (where MA=CH_3_NH_3_, X can be Br, Cl or I). The previous studies have demonstrated that diverse solution-processed function layers deposited on substrate crystals are generally used to fabricate photodetectors because of their transporting of photon-generated charges as well as their blocking of injected current under reverse bias (Wei et al., 2017; Xin et al., 2018; Jariwalad and Hersammc, 2017). To establish p-i-n junctions and decrease the dark current, the function layers based on solution-processed are requested for intrinsic MAPbBr_2.5_Cl_0.5_ PSCs. This sandwich structure consists of electron transporting layer (ETL), intrinsic PSCs and hole transporting layer (HTL). For example, N4,N40-bis(4-(6-((3-ethyloxetan-3-yl)methoxy)hexyl)-phenyl)-N4,N40-diphenylbiphenyl-4,40-diamine (OTPD) and N,N0-bis(3-methylphenyl)-N,N0-diphenylbenzidine (poly-TPD), which transport holes, are regarded as p-type semiconductors. And ETLs such as C60, [6,6]-phenyl-C61-butyric acid methyl ester (PCBM) and 2,9-dimethyl-4,7-diphenyl-1,10-phenanthroline (BCP), which transport electrons, are regarded as n-type semiconductors (Wang and Kim, 2017; Wang et al., 2018). In principle, the conduction band energy of intrinsic PSCs should be lower than n-type layer, so that electrons will move to the conduction band of the function layers. And holes will move to the valence band of the inorganic function layers because PSCs' valence band energy should be higher than p-type layer (Sutherland and Sargent, 2016).

**Figure 1:**
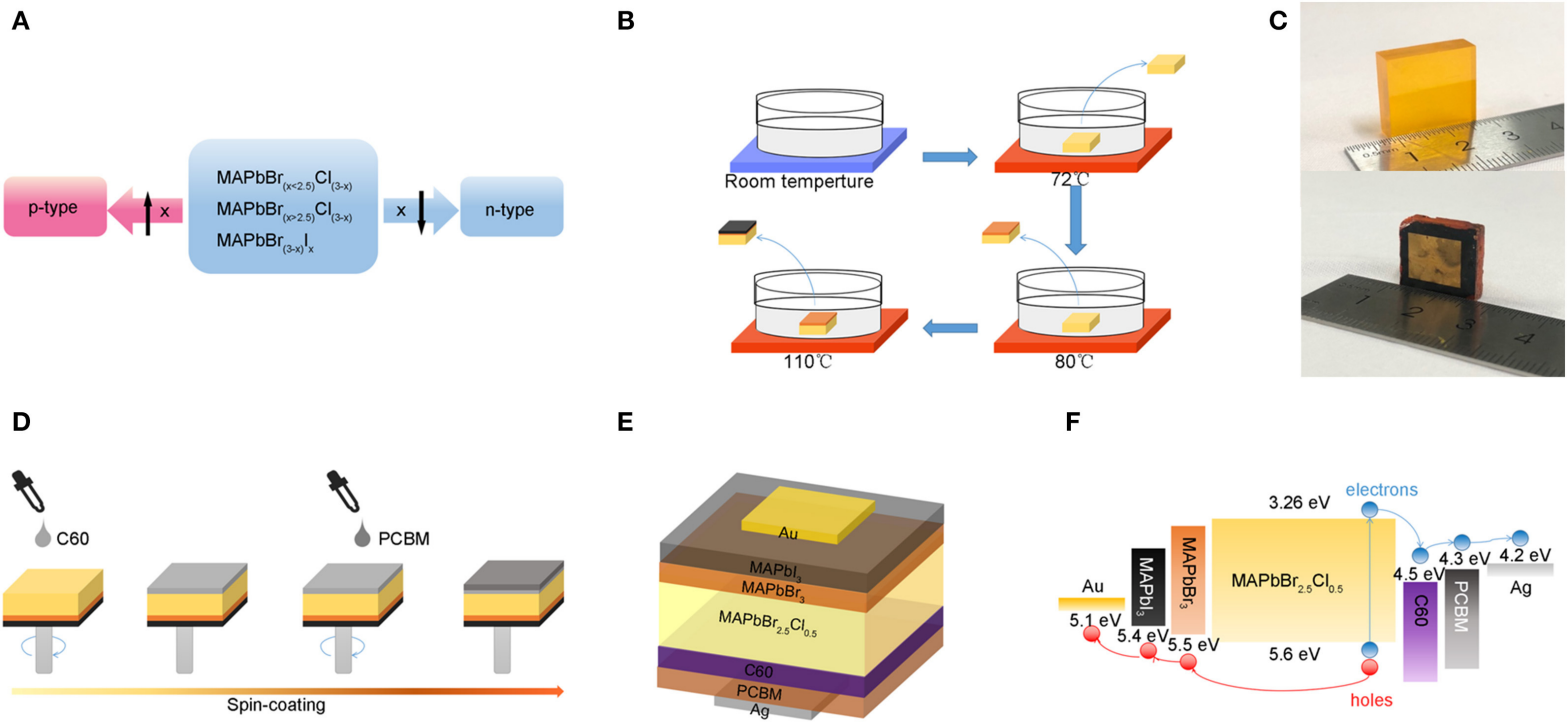
**(A)** Properties of PSCs with different hybrid ratio. **(B)** Processing of epitaxial growth in solution. **(C)** Pictures of MAPbBr_2.5_Cl_0.5_ PSCs and the device. **(D)** Processing of spin-coating method for n-type function layers. **(E)** Device structure of the hybrid perovskite photodetector. **(F)** Energy diagram and the charge transfer behavior of the device under illumination.

However, function layers deposited by spin-coating technique have nanoscale thickness and are easily generating defects, which lead to high traps density and large leakage current (Dong et al., 2015b; Kim et al., 2017). In addition, the function layers have low mobility resulting in difficult transportation of charges. What's more, lattice mismatching is unavoidable between function layers and PSCs substrate. As a result, the devices made by spin-coating method suffer from large inner capacitance due to the accumulation of charges near interface region, which limits the speed of photoelectric response (Xin et al., 2018; Zhao et al., 2014). This work fabricates the photodetector with spin-coated PCBM/C60 films on MAPbBr_2.5_Cl_0.5_PSCs for comparison, the dark current density is 4.24 × 10^−8^ A cm^−2^ at low bias voltage, which is not suitable for weak signal detection. Additionally, the weak tolerance to radiation of MAPbBr_3_ PSC with spin-coated function layers results in added noise for high-energy ray detection has been reported (Xin et al., 2018). We fabrictes the compared device with spin-coated PCBM/C60 films on MAPbBr_2.5_Cl_0.5_PSCs Recently, through changing the halide ratio, such as MAPbI_3−x_Br_x_ and MAPbBr_3−x_Cl_x_, the band gap of OIHPSCs can be modulated from 1.5 to 2.9 eV (Hu et al., 2016; Fang et al., 2015; Jiang et al., 2019). Among these OIHPSCs, MAPbBr_(x<2.5)_Cl_(3−x)_ PSCs, OIHP single crystals become more n-type with x decreasing. On the other hand, MAPbBr_(x>2.5)_Cl_(3−x)_ and MAPbBr_(3−x)_I_x_ PSCs become more p-type with x increasing as shown in Figure 1A (Wang et al., 2014; Jiang et al., 2019).

Therefore, we suppose to employ heteroepitaxial growth technique by solution-processed to form p-i-n junctions. According to the lattice match rule, high quality perovskite layers have to be grown directly on the surface of substrate OIHPSCs in its precursor solution during crystallization process. In this work, we demonstrate that epitaxial perovskite films of high crystallinity could be grown on OIHPSCs. Compared with the spin-coated function layers, epitaxial perovskite films increase the carrier mobility and decrease the trap density. By controlling the time of epitaxial growth in precursor solution, the thickness of perovskite layers can be controlled in micrometer scale, which ensures the devices work stably under high voltage bias. In that case, our perovskite device of centimeter-scale thickness is mainly dedicated to high-energy ray detection such as X-ray detection. Herein, we fabricate the XPD with MAPbBr_2.5_Cl_0.5_ PSCs as substrate crystals. Then epitaxial MAPbBr_3_ mono-crystalline thin films and MAPbI_3_ mono-crystalline thin films replace spin-coated organic function layers as p-type, meanwhile PCBM and C60 films act as n-type. The epitaxial MAPbBr_3_ layers are used as buffer layers to satisfy the lattice matching condition for epitaxial MAPbI_3_ layers and MAPbBr_2.5_Cl_0.5_ PSCs. This XPD has shown low dark current density of 6.97 × 10^−11^ A cm^−2^ at −0.5 V bias, average electrons mobility of 385.9 cm^2^V^−1^s^−1^, high responsivity of 870 mA/W at −100 V bias, average noise current of 1.34 nA Hz^−0.5^ in darkness under −100V bias as well as high X-ray sensitivity of 59.7 μC mGy^−1^ cm^−2^ at −50 V bias.

## Materials and Methods

Materials: Lead iodide (PbI_2_, 99%), lead bromine (PbBr_2_, 98%) and Lead chloride (PbCl_2_, 99%) were purchased from Sigma Aldrich. Methylamine alcohol solution (30–33 wt. % in ethanol) and Hydroiodic Acid (40%) and Hydrobromic Acid solution (55%) were obtained from Macklin. PCBM and C60 were purchased from Aladdin. All commercial products were used as received.

Device fabrication: The precursor is a N,N-dimethylformamide (DMF) solution which dissolves 1M MABr, PbBr_2_ and PbCl_2_ with a ratio of 1:0.75:0.25. After totally reacting and cooling down, the precursor solution were heated in crystallizing dish for 90 h from 50 to 75°C at a rate of 0.3°C h-1 for growth of high-quality MAPbBr_2.5_Cl_0.5_ PSCs. 1M MABr, PbBr_2_ with a ratio of 1:1 are dissolved in a DMF solution for epitaxial growth of MAPbBr3 mono-crystalline thin films at 80°C. And 1M MAI, PbI_2_ with a ratio of 1:1 are dissolved in a γ-GBL solution to grow MAPbI_3_ mono-crystalline thin films at 110°C. The programmable heating control system was realized on IKA-RET control-visc.

Spin-coating method is used to form C60 and PCBM function films respectively on the bottom surface of MAPbBr_2.5_Cl_0.5_ PSCs at 2500 r.p.m for 20s. C60, PCBM and sliver electrode solution were deposited on the opposite face by thermal evaporation in vacuum. MAPbBr_3_ mono-crystalline thin films, MAPbI_3_ mono-crystalline thin films and gold electrode were deposited on opposite surfaces of PSCs.

Characterization: SEM images were taken with a Quanta 200 FEI (USA). XRD patterns were obtained by X'TRA (Switzerland). PL spectra were measured by UV–vis spectroscopy (Lab Tech Bluestar, USA).

Device Performance Measurements: Keithley 2410 was used as the voltage source to measure J-V curves. Noise of the device was characterized with a 450 nm LED at 10 Hz and Keithley 2410 as voltage source. The 460 nm light source is obtained from the F5 LED of GEESLED Company. A 355 nm pulsed Nd:YAG laser with 6 ns pulse width at 20 Hz was used as the illumination source and response time was measured using an Agilent oscilloscope with a Keithley 2410 as the voltage source. The X-ray source is obtained from PERLOVE Medical Company and the X-ray dose rate was obtained by a commercial dosimeter (FJ-347A, China).

## Results and Discussion

PSCs used in this work are grown by variable temperature crystallization (VTC) method which has been previously reported. (Stoumpos et al., 2013; Maculan et al., 2015) The fabrication procedure of MAPbBr_2.5_Cl_0.5_ PSCs and epitaxial perovskite mono-crystalline films is depicted in Figure 1B. As illustrated in Figure 1C, the size of MAPbBr_2.5_Cl_0.5_ PSC is approximately 18 mm × 18 mm in width and length, as well as 5 mm in thickness. The MAPbBr_2.5_Cl_0.5_ PSC is dipped into MAPbBr_3_ and MAPbI_3_ perovskite precursor solution respectively at optimum growth temperature. Bragg equation is used to calculate the lattice constants of MAPbBr_2.5_Cl_0.5_, MAPbBr_3_ and MAPbI_3_ (Jiang et al., 2019; Ji et al., 2018). Additionally, X-ray diffraction patterns of each single crystal are shown in Figure S1 for comparison. And the lattice constants of MAPbBr_2.5_Cl_0.5_, MAPbBr_3_ and MAPbI_3_ are 5.87Å, 5.93 Å and 6.23 Å, respectively. Besides, the lattice mismatching rates of MAPbBr_2.5_Cl_0.5_-MAPbBr_3_ and MAPbBr_3_-MAPbI_3_ are calculated to be only 1.01 and 4.81%. As a result, MAPbBr_3_ and MAPbI_3_ mono-crystalline thin films are formed on the surface of the MAPbBr_2.5_Cl_0.5_ PSCs in sequence. After cutting and polishing process, the epitaxial mono-crystalline thin films exist only on the top of substrate. As shown in Figure 1D, C60 and PCBM films, which act as n-type materials, are deposited on the bottom of MAPbBr_2.5_Cl_0.5_ PSCs via spin-coating method. The gold and silver electrodes are deposited on the top and bottom surfaces of the PSCs by thermal evaporation in vacuum. Figure 1E depicts the Au-MAPbI_3_-MAPbBr_3_-MAPbBr_2.5_Cl_0.5_-C60-PCBM-Ag structure of our photodetector. The energy diagram and the charge transfer behavior of this XPD with incident light from silver electrode are shown in Figure 1F. These photogenerated electrons and holes are transmitted by ETLs and HTLs and then are collected separately by the electrodes under external electric field. Moreover, C60 and PCBM films can block holes while MAPbI_3_ and MAPbBr_3_ mono-crystalline thin films block electrons injected by voltage source, which mainly benefit dark current (Bao et al., 2017).

**Figure 2:**
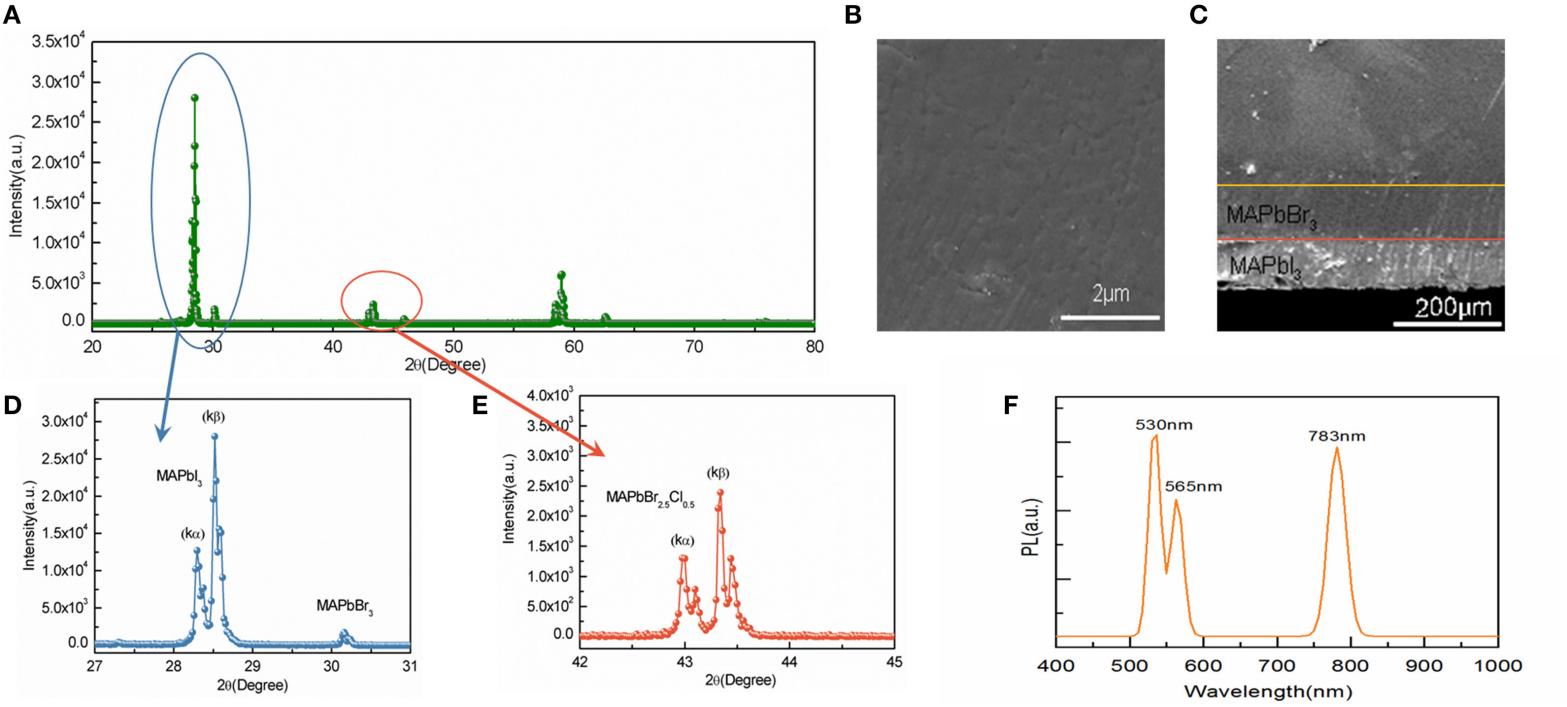
**(A)** X-ray diffraction (XRD) pattern of epitaxial device. **(B)** Scanning electron microscope (SEM) image of the epitaxial MAPbI_3_ film surface. **(C)** SEM image of the cross section of epitaxial films on substrate. **(D)** XRD pattern with Bragg angle from 27 to 31°. **(E)** XRD pattern with Bragg angle from 42 to 45°. **(F)** Photoluminescence (PL) spectra of the perovskite photodetector (with excitation wavelength of 450 nm).

As shown in Figure 2A, X-ray diffraction (XRD) is used to characterize the crystallinity of MAPbBr_3_ films and MAPbI_3_ films on the surface of MAPbBr_2.5_Cl_0.5_ PSCs. Scanning electron microscope (SEM) characterization of the surface of MAPbI_3_ mono-crystalline thin films layer on MAPbBr_3_ mono-crystalline thin films is shown in Figure 2B. Moreover, Figure 2C illustrates the cross-sectional view of the device in which the thickness of each epitaxial crystal layer is clearly visible, with approximately 110 μm of MAPbBr_3_ film and 100 μm of MAPbI_3_ film. Additionally, Energy Dispersive X-Ray (EDX) spectra of substrate and each layer are illustrated in Figure S1 to confirm the layers' thickness. Herein, it can be concluded by our experiments that epitaxial mono-crystalline thin films based on lattice matching have considerable thickness, excellent crystallinity (Pellegrino et al., 2015). Additionally, Figure 2D illustrates that the epitaxial films deposited on structure have a clear MAPbI_3_ peak with full width at half-maximum (FWHM) of 0.105° and this strong peak is separated into kα and kβ peaks, which confirms high crystallization quality. In addition, there is a MAPbBr_3_ peak with FWHM of 0.062°. Similarly, as shown in Figure 2E, the FWHM of MAPbBr_2.5_Cl_0.5_ is 0.112°. For comparison, the XRD patterns of each single crystal are shown in Figure S2.

Figure 2F shows the Photoluminescence (PL) spectra (excited by 450 nm) of MAPbBr_2.5_Cl_0.5_ PSC substrate with epitaxial growth of MAPbBr_3_ and MAPbI_3_ mono-crystalline thin films. Three emission peaks are obtained at wavelength of 530 nm, 565 nm and 783 nm respectively with a LED excitation at 450 nm. In addition, the PL spectra of each single crystal is provided in Figure S3. For comparison, the PL peaks of this heterostructure device obviously become more red-shifted than those of perovskite single crystals. According to previous literatures, the soft and dynamic crystal lattice of halide perovskites are attributed to the low energy barrier for perovskite crystal formation, which easily leads to solid-state ion migrations in the adjacent perovskite lattice (Shewmon et al., 2016; Pan et al., 2018; Zhang et al., 2019). During the process of epitaxial growth in this work, the MAPbBr_2.5_Cl_0.5_-MAPbBr_3_ and the MAPbBr_3_-MAPbI_3_ perovskite heterostructures are fabricated respectively where chloride-bromide and bromide-iodide interdiffusion across the perovskite heterojunction occur which leads to the red-shifted of PL peaks.

**Figure 3:**
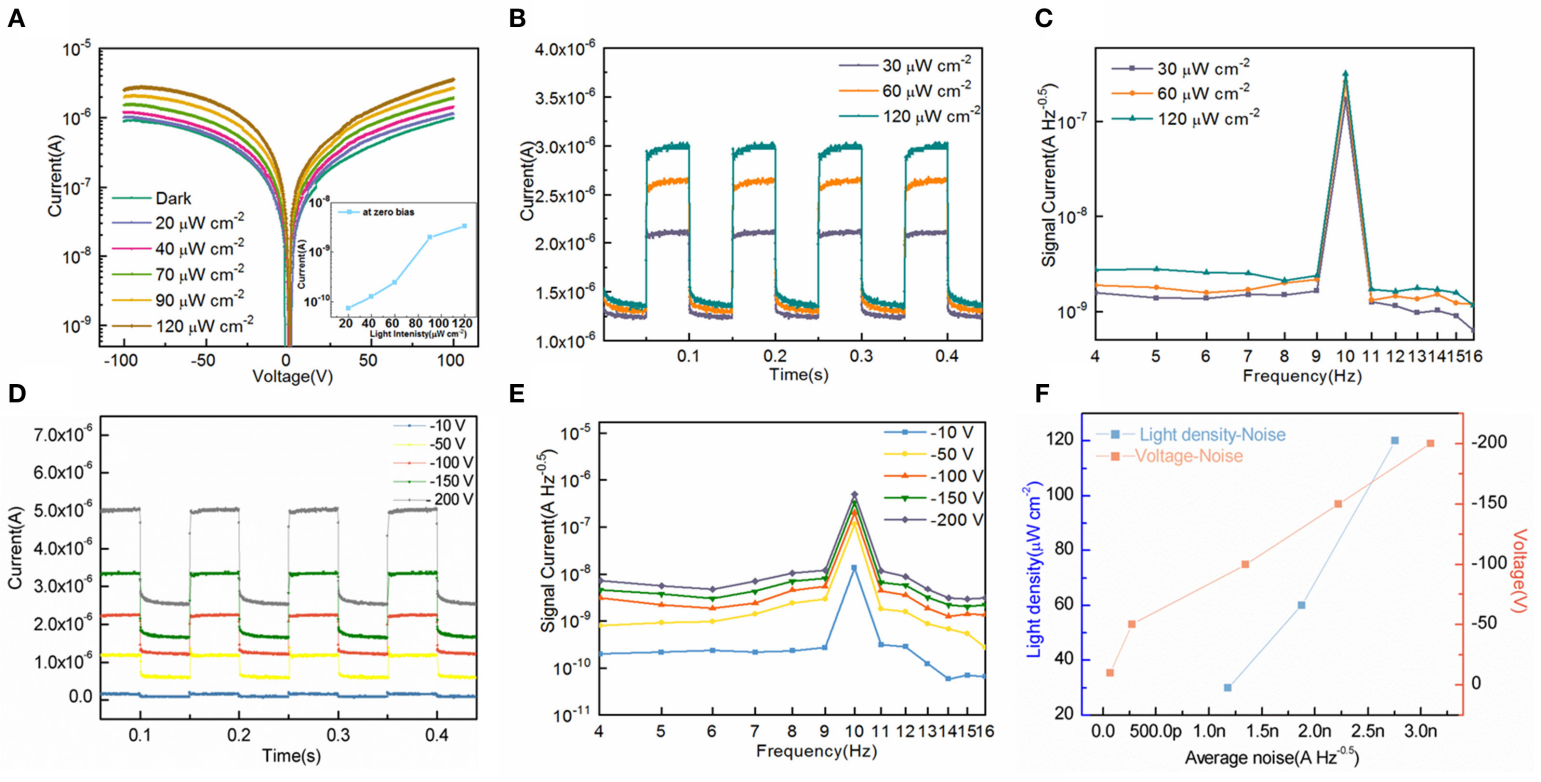
**(A)** J-V curve and photocurrents at zero bias of the device under different illumination density. **(B)** Temporal response of the device under different illumination density at 460 nm. **(C)** Signal current as a function of frequency under different illumination density at −100 V bias. **(D)** Temporal response of the device under different voltage bias at 460 nm. **(E)** Signal current as a function of frequency under different voltage bias. **(F)** Plots of light density and voltage vs. average noise.

To characterize the electric properties of this photodetector for visible light, a white light emitting diode with adjustable illumination density is used to radiate gold electrode of the device. Due to the dark current denisity of photodetectors should be as low as possible in order to recognize weak signal current from noise (Nakamura et al., 2001; Li et al., 2020). The current density-voltage curves of our device under the sweep voltage from −100 V to 100 V at different illumination denisty are shown in Figure 3A. Thanks to the epitaxial mono-crystalline films working as blocking layers, the dark current density of this device is fairly low of 6.97 × 10^−11^ A cm^−2^ at −0.5 V bias, which is one tenth of the CH_3_NH_3_PbI_3_ perovskite device reported before only with thick PCBM (50 nm)/C60 (130 nm) layer as the ETL (Lin et al., 2015). What's more, we fabricate Device A with spin-coated PCBM/C60 films on the MAPbBr_2.5_Cl_0.5_ substrate as the ETLS for comparison. The picture and J-V curve of Device A are shown in Figure S4. The dark current density of Device A at −0.5 V bias is 4.24 × 10^−8^ A cm^−2^, which is three orders of magnitude higher than our photodetector based on epitaxial mono-crystalline films. When the illumination density increases from 20 μWcm^−2^ to 120 μWcm^−2^, the photocurrent density increase from 1.81 μA cm^−2^ to 5.12 μA cm^−2^ at −50 V bias. Besides, the device could work at zero-bias as shown in the inset due to the in-built electrical field.

Figure 3B illustrates the temporal response of photodetectors based on epitaxial crystal layers with increased density of 460 nm illumination at 10 Hz. In order to get the signal noise at low frequency of the device, the Fast Fourier Transform (FFT) method is used to transfer photocurrent density vs. time into signal current density vs. frequency (Dong et al., 2015b). And we analyze the signal current density ranges from 1 to 16 Hz, as shown in Figure 3C. The noise current at low frequency increases approximately from 0.62 to 1.54 nA Hz^−0.5^ under current 30 μW cm^−2^ illumination density. With light density increasing from 30 to 120 μW cm^−2^, the noise current increases from 1.18 to 2.80 nA Hz^−0.5^ at −100 V bias.

Due to X-ray detection requires large voltage bias to collect charges induced in millimeter-thickness PSCs, here we characterize the noise current when the voltage bias varies from −10 V to −200 V. Similar with Figures 3B,D shows the temporal response at 10 Hz with different voltages under 120 μW cm^−2^ illumination density at 460nm. The average noise current increases from 68.7 pA Hz^−0.5^ to 3.24 nA Hz^−0.5^ in darkness and increases from 0.25 nA Hz^−0.5^ to 11.9 nA Hz^−0.5^ under illumination as shown in Figure 3E. In conclusion, the function of light density and voltage vs. average noise is shown in Figure 3F. This device with epitaxial mono-crystalline films is an excellent candidate for X-ray detection in terms of their low signal noise performance under high voltage bias (Kasap et al., 2011).

What's more, Responsivity (R), the ratio of photocurrent density and incident illumination density, indicates the efficiency of photodetectors responds to optical signal. And the formula is

$$\begin{array}{l} \text{R}=\frac{{\text{J}}_{\text{light}}-{\text{J}}_{\text{dark}}}{\text{P}} \end{array}$$

where **J**_**light**_ is photocurrent denisty, **J**_**dark**_ is dark current denisty and **P** is incident illumination denisty (Dou et al., 2014). The area of photodetector is 25 mm^2^. As illustrated in Figure 3D, R can be calculated to approximately 87 mA/W at −100 V and 23 mA/W at −50 V bias with 120 μWcm^−2^ illumination at 460 nm.

**Figure 4:**
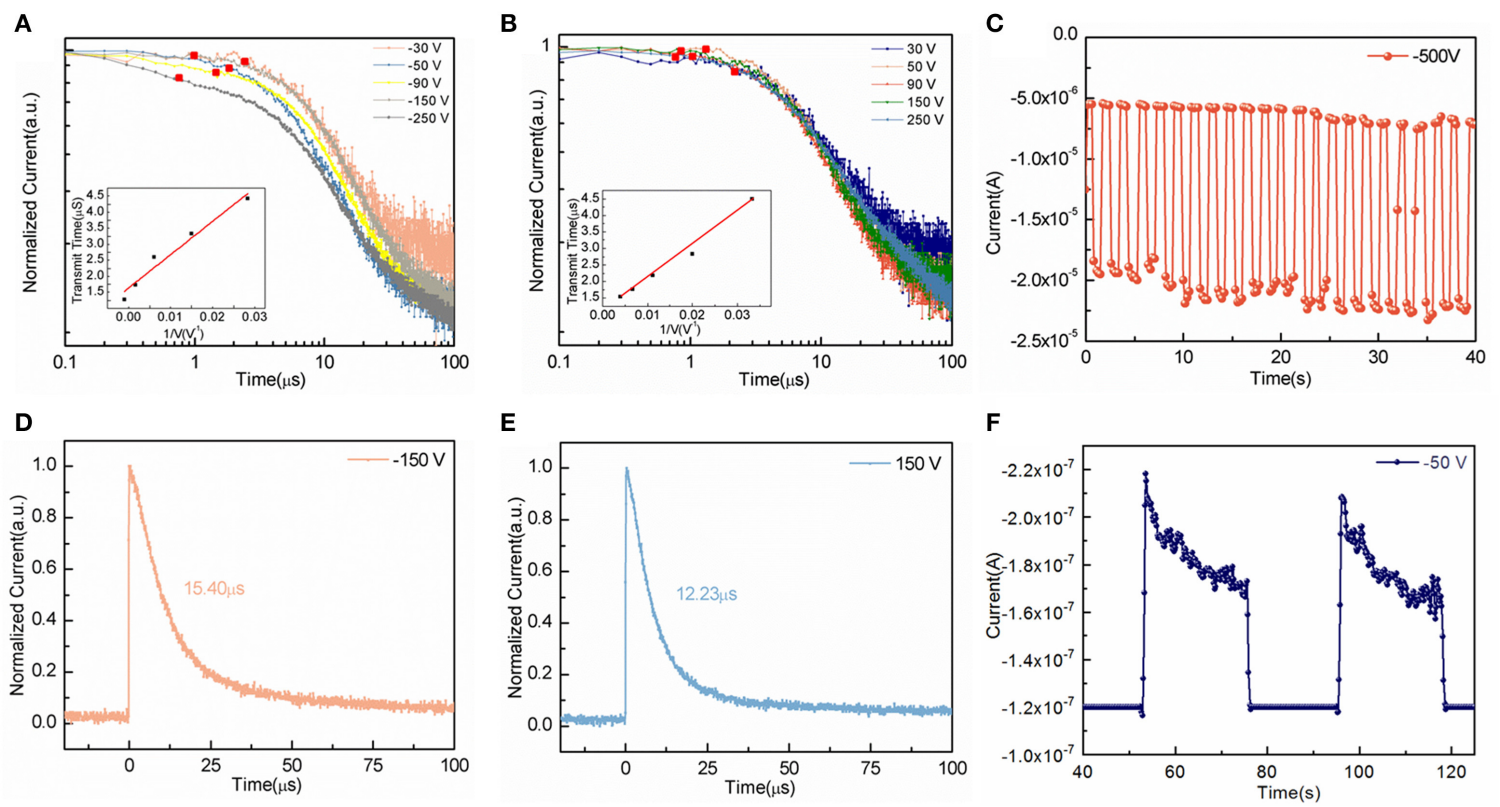
**(A)** Electrons mobility caculated by time-of-flight (TOF) method. **(B)** Holes mobility caculated by TOF method. **(C)** Long-term transient response of the device at −500 V bias. **(D)** Response time of the device at −150 V bias. **(E)** Response time of the device at 150 V bias. **(F)** Transient response to X-ray at 40 kV and 1 mA.

Figures 4A,B provide the time of flight (TOF) curves to measure the carrier mobility of semiconductor material. According to

$$\begin{array}{l} \mu =\frac{{\text{L}}^{2}}{{\text{T}}_{\text{tof}}{\text{V}}_{\text{s}}} \end{array}$$

the carrier mobility **μ** can be directly calculated once we get the distance **L** and voltage bias **V**_**s**_ between two electrodes and the carrier transit time **T**_**tof**_. (Huang et al., 2015; Wei et al., 2016) As depicted in Figure 4A, the average mobility of electrons is calculated to 385 cm^2^V^−1^s^−1^ under reverse voltages. Similarly, Figure 4B shows the average holes mobility of 415 cm^2^V^−1^s^−1^ under forward voltages. The mobility here is larger than the those of devices based on PSCs results from the decreased dislocation between epitaxial grown films and lattice matched substrate (Dong et al., 2015a; Ji et al., 2018).

Figure 4C illustrates stable dark current and photocurrent of the photodetector under high work voltage of −500 V bias at 460 nm. The long-term stability of the device benefits from epitaxial mono-crystalline films replacement of function layers (Wang et al., 2019). As shown in Figures 4D,E, the response time of device is 15.40 μs under reverse bias and 12.23 μs under forward bias, which is 3 orders of magnitude faster than those of photodetectors based on perovskite polycrystalline thin films (Liu et al., 2019; Su et al., 2019).

The temporal X-ray response of our device is illustrated in Figure 4F. With X-ray off, the dark current density is 480 nA cm^−2^ at −50 V bias. And the photocurrent density reaches 880 nA cm^−2^ to X-ray at 40 kV and 1 mA with dose rate of 6.7 μGys^−1^. The sensitivity of our device is 59.7 μC mGy^−1^ cm^−2^ which is almost five times higher than X-ray detectors based on polycrystalline perovskite reported recently (Kim et al., 2017).

## Conclusion

In summary, this work demonstrates a perovskite photodetector for X-ray detection fabricated with epitaxial mono-crystalline films as electron-blocking layers. Due to lattice matching, we fabricates the device with epitaxial layers of high crystallinity and considerable thickness, which are characterized by XRD and SEM. Furthermore, our X-ray photodetector shows low dark current density of 6.97 × 10^−11^ A cm^−2^ at −0.5 V bias, high electrons mobility of 385.9 cm^2^V^−1^s^−1^, high responsivity of 870 mA/W at −100 V bias and remarkable X-ray sensitivity of 59.7 μC mGy^−1^ cm^−2^ at −50 V bias. This work offers a path of heteroepitaxial mono-crystalline films acting as electron-blocking layers for solution-processed photodetectors based on OIHPSCs.

## Data Availability Statement

The raw data supporting the conclusions of this article will be made available by the authors, without undue reservation.

## Author Contributions

YX, YL, and YP grew the perovskite single crystals. YX did the epitaxial experiments. YX and YP did the measurements. XW and YX analyzed these results. YX wrote this manuscript. All authors make comments on the manuscript.

## Conflict of Interest

The authors declare that the research was conducted in the absence of any commercial or financial relationships that could be construed as a potential conflict of interest.
