# Supplementary material for: Perovskite Photodetectors Based on p-i-n Junction With Epitaxial Electron-Blocking Layers
Source: Front Chem. 2020 Sep 15;8:811. doi: 10.3389/fchem.2020.00811 (PMC7522216; doi:10.3389/fchem.2020.00811)
Supplement: Supplementary file 1 [file Data_Sheet_1.PDF]

frontiers

Supplementary Material

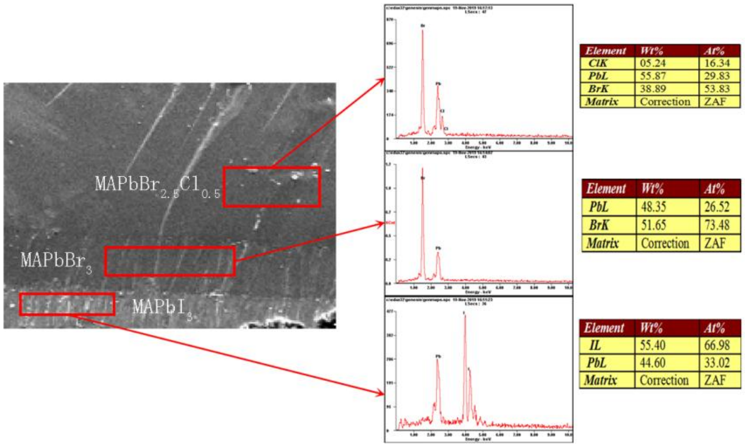

Supplementary Figure S1. Energy-dispersive X-ray (EDX) spectrum of the device at different PSCs layers with different halide element.

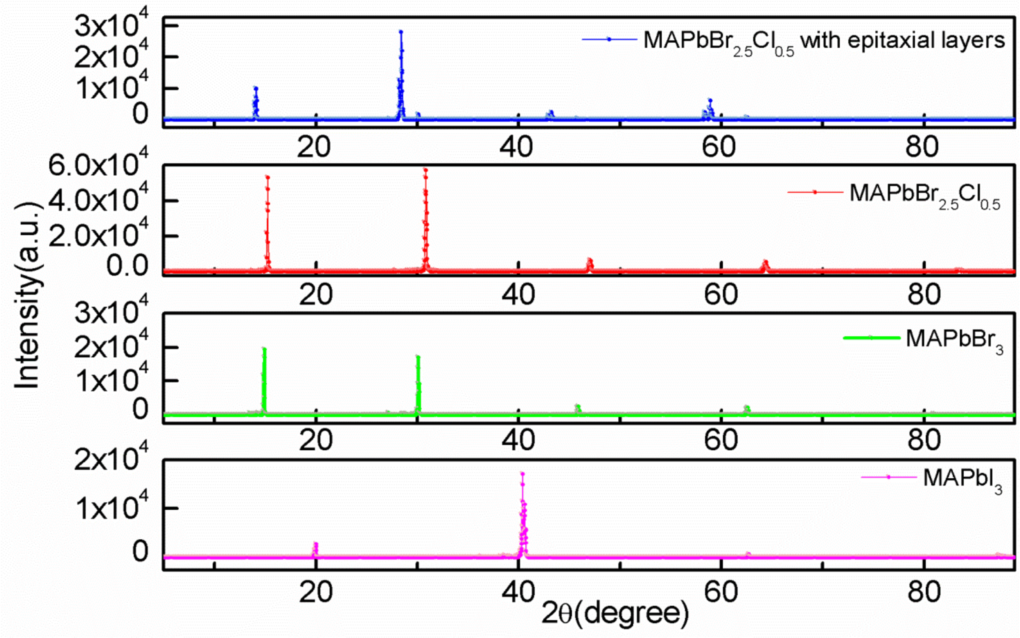

Supplementary Figure S2. X-ray diffraction (XRD) patterns of epitaxial device, $MAPbBr_{2.5}Cl_{0.5}$ PSCs, $MAPbBr_3$ PSCs and $MAPbI_3$ PSCs.

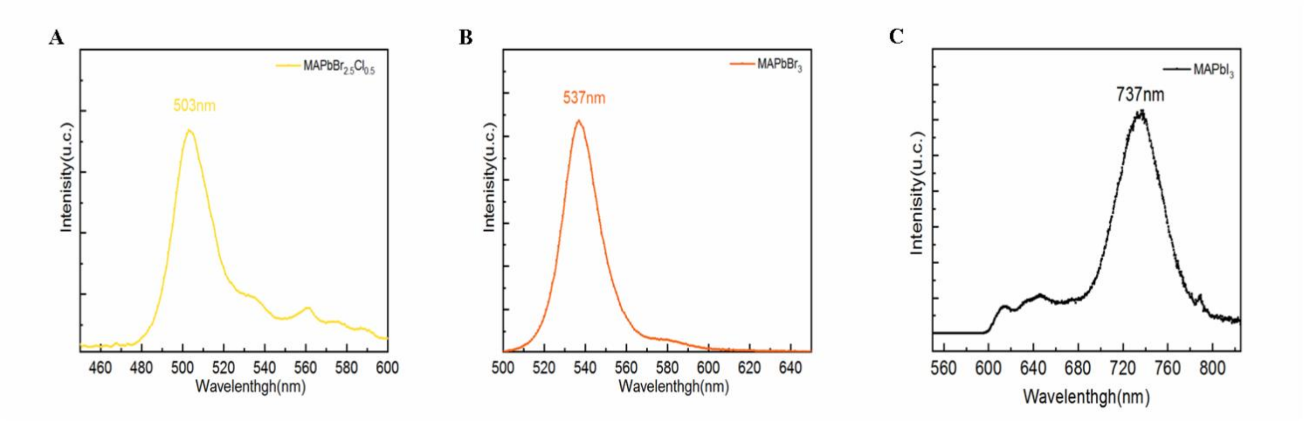

Supplementary Figure S3. Photoluminescence (PL) spectra of each single crystal for comparison **(A)** $MAPbBr_{2.5}Cl_{0.5}$ PSCs **(B)** $MAPbBr_3$ PSCs **(C)** $MAPbI_3$ PSCs.

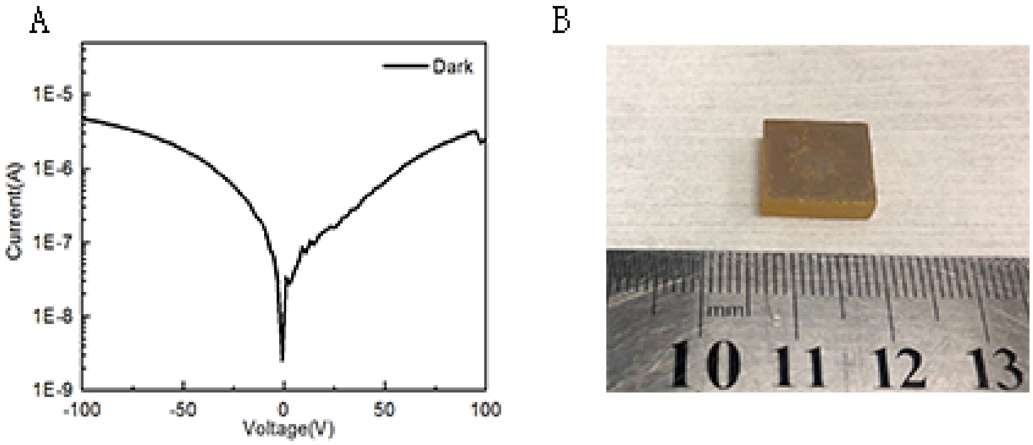

Supplementary Figure S4. (A) J-V curve of Device A **(B)** Picture of Device A
